# Cervical cancer perceived behavioral risk factors using logistic regression technique

**DOI:** 10.7555/JBR.39.20250047

**Published:** 2026-05-21

**Authors:** I. M. Elzein, Ashraf Chamseddine, Ahmad Eltanboly, Adam Elzein

**Affiliations:** 1 Department of Electrical Engineering, College of Engineering and Technology, University of Doha for Science and Technology, Doha 24449, Qatar; 2 Department of Environmental Health and Safety, University of Doha for Science and Technology, Doha 24449, Qatar; 3 Faculty of Science, Galala University, New Galala City, Suez Governorate 43111, Egypt; 4 Department of Medicine, Medical University of Lodz, Lodz 90-419, Poland

**Keywords:** principal component analysis, cervical cancer, behavioral risk factors, logistic regression, feature engineering, regularization

## Abstract

Cervical cancer represents a considerable global health challenge, mainly because of ineffective screening programs in low-income countries. The current study aimed to forecast cervical cancer incidence by analyzing behavioral risk factors through logistic regression, employing feature engineering techniques such as principal component analysis (PCA). PCA successfully condensed the dataset into ten principal components, capturing 89% of the variance, while stratified K-fold cross-validation ensured a balanced representation of classes. With the application of L1 regularization, the logistic regression model achieved an accuracy of 97.2%, an area under the curve (AUC) of 98.1%, an F1 score of 97.2%, a specificity of 96.1%, and a log loss of 0.17. The performance of the models was comparatively evaluated, and the results revealed that the logistic regression model achieved the highest accuracy of 97.2% compared with decision trees at 93.33%, random forest at 93.33%, XGBoost at 93.33%, naive Bayes at 91.67%, and non-regularized logistic regression at 87.55%. This research underscores the importance of early prediction of cervical cancer based on behavioral risk factors and suggests a robust, easily implementable workflow to improve classification accuracy. Future research should concentrate on refining these predictive tools to overcome social and behavioral barriers to prevention, particularly within underserved populations.

## Introduction

One of the main factors contributing to the significantly higher prevalence of cervical cancer in low-income nations is the lack of efficient screening programs for identifying and treating precancerous conditions^[1]^. In 2018, approximately 569000 new cases of cervical cancer were diagnosed worldwide^[2]^. Early detection of cervical cancer can be facilitated through screening and diagnostic techniques^[3]^, such as acetic acid visual inspection, Pap smears, human papillomavirus testing, and colposcopy, which can reduce the incidence through timely intervention^[4]^.

Behavior plays a significant role in estimating the risk of cervical cancer^[5]^. Various behavior-related theories and models, such as the Health Belief Model (HBM), Protective Motivation Theory (PMT), Theory of Planned Behavior (TPB), and Social Cognitive Theory (SCT), are commonly used to study behavior in relation to health^[6]^.

As shown in ***Table 1***, a comparison is made between previous studies that focused on predicting cervical cancer based on behavioral risk factors. Kumar *et al*^[7]^ used three machine learning models (Random Forest, Decision Tree, and XGBoost) on a dataset containing 72 records and 19 attributes, achieving an accuracy of 93.33% for all classifiers. The analysis focused mainly on data preprocessing and exploratory data analysis, which helped select the most important features, but the method was complex and relied heavily on exploratory data analysis. Sobar *et al*^[8]^, who also worked on behavioral risk factor datasets, employed Naive Bayes and logistic regression classifiers, achieving accuracies of 91.67% and 87.55%, respectively. In another study, Asadi *et al*^[9]^ used a dataset containing 145 patients with 23 attributes and applied machine learning classification algorithms, including Support Vector Machines (SVMs). Their results achieved an accuracy of 79%, a precision of 67%, and an area under the curve (AUC) of 85%.

**Table 1:** Comparison of previous studies on machine learning models, evaluation metrics, and main features

Authors	Machine learning models	Evaluation metrics	Main features
Kumar *et al*^[7]^	Decision Tree, Random Forest, Logistic regression	Accuracy: 82.0%, 85.1%, 80.2%	Data preprocessing, exploratory data analysis
Sobar *et al*^[8]^	Naïve Bayes, Logistic regression	Accuracy: 91.67%, 87.55%	Publicly available dataset, risk factor based on psychological theory
Deng *et al*^ [10 ]^	XGBoost, SVM, Random Forest	RF sensitivity: 95.1% (Hinselmann), XGBoost sensitivity: 94.3% (Hinselmann); XGBoost reaches 98.3% for Cytology	Synthetic Minority Oversampling Technique (SMOTE), Borderline-SMOTE, and top five risk factors selection
Nithya *et al*^[ 11]^	C5.0, RPART, SVM, KNN	Accuracy: 97%, 96%, 88%, 89%	Feature selection techniques, a variety of classifiers
Tseng *et al*^[12]^	SVM, C5.0, Extreme Learning Machines	Accuracy: 96.28%, 84.04%, 96.28%	Identification of significant risk factors for predicting recurrence-proneness of cervical illness
Mohi Uddin *et al*^[13 ]^	Random Forest, SVM, Linear regression	Accuracy: 98.91%, precision: 97.81%, F1-score: 0.9889, AUC: approximately 1.0	Rely on tissue slide analysis and risk factors instead of early prediction
Wu *et al*^[15]^	Support Vector Machine (SVM)	Accuracy: 93.97%, sensitivity: 98.73%	Decreased the number of significant predictors, reduced computing cost

Deng *et al*^[10]^ used XGBoost, SVM, and Random Forest to assess data on cervical disease. The dataset was sourced from the University of California, Irvine (UCI) Machine Learning Repository, which included 32 risk factors and four goal variables from 858 individuals' clinical histories. To deal with the dataset's imbalance, the Borderline Synthetic Minority Over-sampling Technique (Borderline-SMOTE) was applied. The use of SMOTE in the preprocessing stage and the selection of the top five risk factors in this context helped enhance the results. The use of the SMOTE technique for preprocessing was necessary because of the dataset's characteristics, such as imbalanced data, which may have limited the approach. Despite the promising outcomes obtained from the application of XGBoost, SVM, and Random Forest, it can be inferred that the initial dataset might not have been suitable for these techniques without additional preprocessing^[10]^.

Nithya *et al*^[11]^ used machine learning to investigate the risk factors for cervical cancer. The dataset included 858 rows and 27 features. Using 10-fold cross-validation, C5.0 achieved an accuracy of 97%, followed by Random Forest at 96.9%, rpart at 96%, and both SVM and KNN at 88%. Tseng *et al*^[12]^ used SVM and C5.0 to discover significant risk factors for predicting the recurrence of cervical cancer. SVM and C5.0 achieved accuracies of 68.00% and 96.00%, respectively. They employed Extreme Learning Machines (ELMs) to identify important risk factors for forecasting the likelihood of cervical cancer recurrence. Incorporating ELM into the models could pose challenges in terms of interpretability. Understanding the significant risk factors identified for predicting the likelihood of cervical cancer recurrence was challenging because of the interpretability issues associated with these models^[13]^.

It is important to note that the most significant gap identified from the literature review is the neglect of behavioral risk factors as predictors of cervical cancer^[14]^. Although several studies examined feature selection, exploratory data analysis, and information preprocessing approaches, they did not specifically address the prediction of cervical cancer based on behavioral risk factors^[16]^. Additionally, some studies have reported low accuracy because of the absence of a preprocessing stage^[17]^ or have failed to include a proper literature evaluation^[17]^. Furthermore, a shortage of studies on early prediction of cervical cancer based on behavioral risk factors has been observed.

The primary objective of the current study was to address the deficiencies in existing literature by developing a robust algorithm that uses optimized hyperparameters derived from behavioral risk factors, which may help enhance the early detection of cervical cancer^[19–20]^. Specifically, this investigation aimed to formulate a reliable and efficient system for the early prediction of cervical cancer, emphasizing behavioral risk factors that have frequently been overlooked in prior research. By merging thoughtful feature selection, dimensionality reduction, and meticulous model tuning, the proposed methodology simplifies the diagnostic process while enhancing accuracy. It also incorporates stratified cross-validation to ensure that results are balanced throughout the dataset.

## Materials and methods

### Description of the dataset

The dataset for this research contains 72 instances and includes 19 attributes with two classes. The term "ca-cervix"^[21]^ refers to the condition of cervical cancer, where a value of zero indicates the absence of cervical cancer and a value of one indicates the presence of cervical cancer^[22]^.

### Methodology

**Figure 1:**
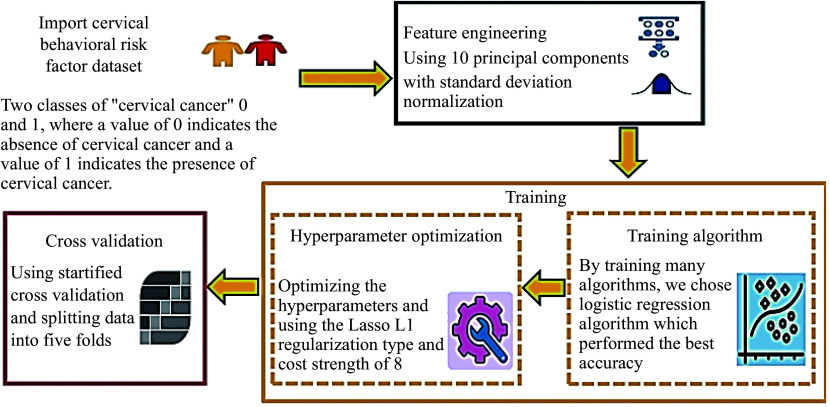
The proposed methodology.

An early cervical cancer prediction framework was established by using behavioral risk factors with the assistance of a data mining tool. As shown in ***Fig. 1***, four main stages were executed using a data mining tool. Initially, the dataset was imported, followed by the application of preprocessing techniques^[23]^. Next, the logistic regression algorithm was selected, hyperparameters were optimized, and subsequently, the algorithm was trained using the stratified K-fold cross-validation (SKCV) technique (***Table 2***). To assess the effectiveness of the model, we carried out SKCV with five folds.

**Table 2:** The process of the main algorithm used in the proposed work to illustrate the workflow of the whole process

The general process of the main algorithm
1. Datasets from the University of California, Irvine Machine Learning Repository were imported.
2. Feature engineering and preprocessing: Check for any missing values; Verify the int64 data format; Use principal component analysis to identify the top 10 principal components; Normalize a subset of the features using standard deviation normalization.
3. Select the logistic regression approach as a suitable option for this application.
4. Model learning: Use Lasso L1 regularization with a cost strength of 8 to optimize the hyperparameters; The sigmoid function was used to map the output of the logistic regression to a probability value between 0 and 1.
5. Evaluation: Use classification accuracy, area under the curve, precision, recall, specificity, log loss, and F1-score to evaluate the performance of this model.
Abbreviation: Lasso, Least Absolute Shrinkage and Selection Operator.

**Figure 2:**
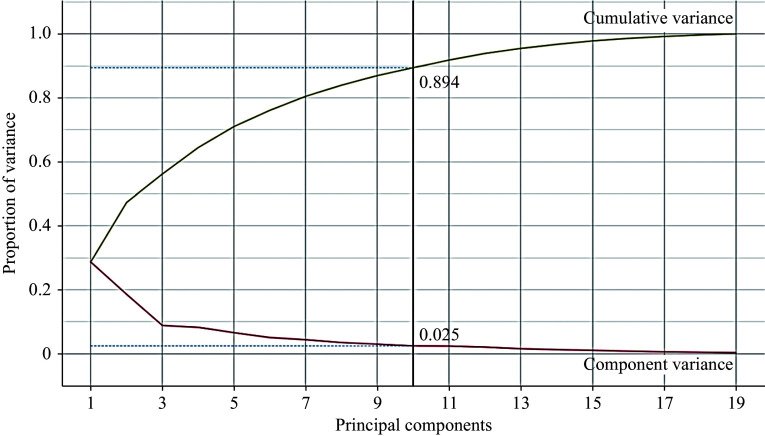
Degree of variance covered by the best principal component number.

**Figure 3:**
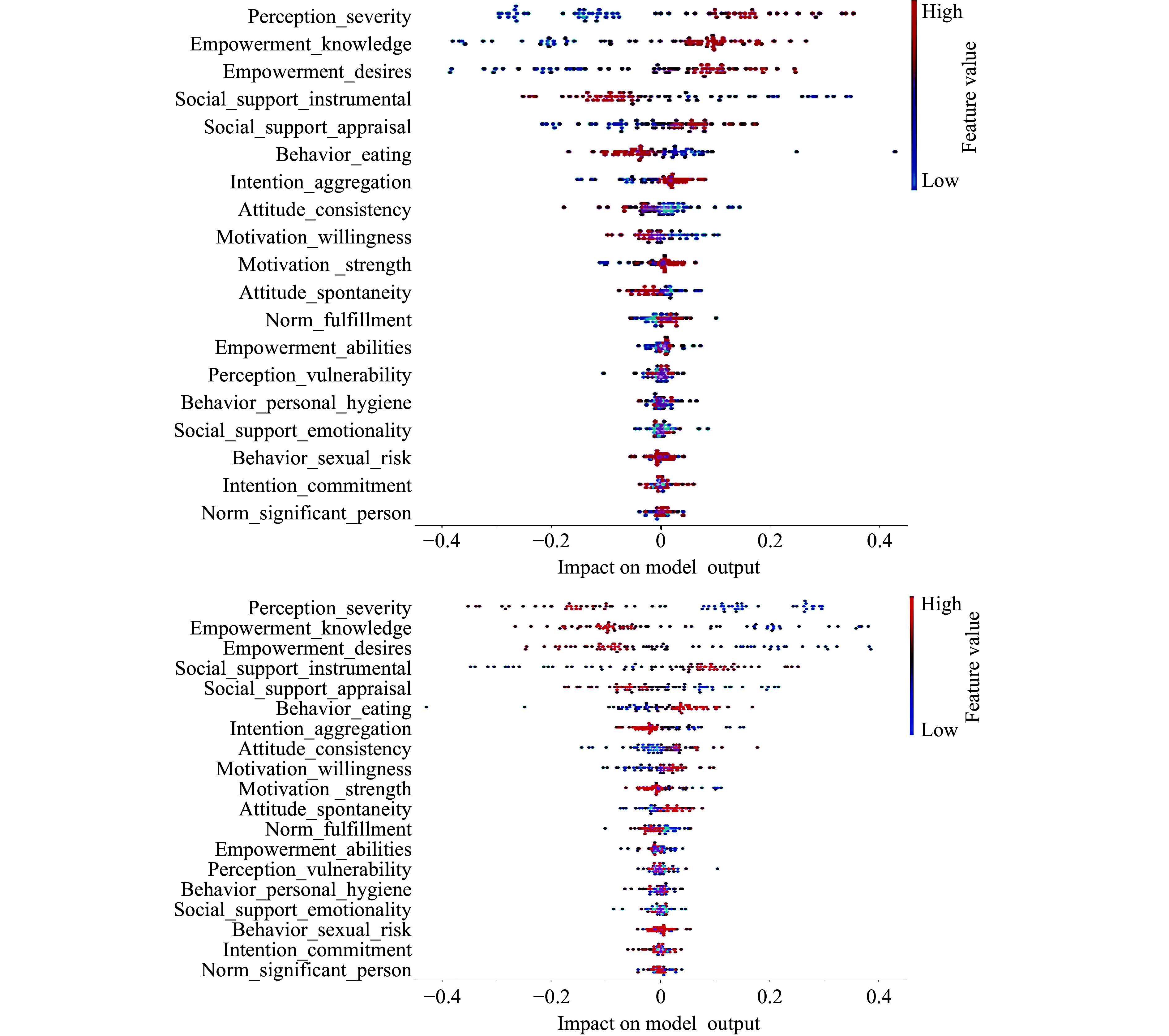
The SHAP library's Explain Model widget. The upper panel depicts a Shapley Additive Explanations (SHAP) summary plot of class 1 (presence of cervical cancer) feature importance, while the lower panel indicates feature impact for class 0 (absence of cervical cancer). Both plots show the influence of individual features on the final prediction output of the model.

The dataset was imported with no missing values, and all attributes, including the class variable, were represented as 64-bit integer values, thereby eliminating the need for encoding^[24]^. Feature engineering and preprocessing were performed in the second stage to enhance the outcome. Principal component analysis (PCA) was applied for feature reduction, transforming correlated features into linearly uncorrelated components. The selected features were condensed into ten principal components with standard deviation normalization, covering approximately 89% of the variance in the features, as shown in ***Fig. 2***. This approach optimized the quality of the results^[25]^. The scree plot, exhibited by the PCA widget (***Fig. 2***), is a significant tool that reveals the variation accounted for by each principal component^[26]^. With a maximum correlation coefficient of 0.85, the application of feature selection techniques was rendered unnecessary^[15]^. The model diagram (***Fig. 3***) illustrates the significant contribution of processed features to the output model. The "Explain Model" widget from the Shapley Additive Explanations (SHAP) library details classification models and the influence of each feature on them (***Fig. 3***). The "Explain Model" widget employs SHAP values to shed light on the mechanisms behind the model's predictions^[27]^. This widget enables users to comprehend the elements that affect the model's predictions and to gain insights into its operational dynamics. Additionally, this tool aids users in deciphering complex models and in recognizing potential biases or challenges within the decision-making framework^[25]^.

The logistic regression algorithm was selected because it was deemed the most suitable for the problem at hand^[8]^. Several algorithms were evaluated; however, all of them yielded an accuracy below 93%. The existing method achieved an accuracy of 93%, which served as the benchmark for our research. In this stage, hyperparameters were optimized to improve the results. The Least Absolute Shrinkage and Selection Operator (Lasso) with L1 regularization was used, and the cost strength was set to eight, which significantly enhanced the logistic regression accuracy. In the final stage, SKCV with five folds was employed to evaluate the results, and the algorithm was trained.

### Configuration of comparative models

Besides the proposed logistic regression model framework, benchmarking was conducted using three widely used machine learning classifiers: Decision Tree, Random Forest, and eXtreme Gradient Boosting (XGBoost). Their inclusion provides baseline comparisons for the proposed logistic regression model in terms of predictive performance and computational cost. Using Python 3.14, we implemented Decision Tree and Random Forest *via* Scikit-learn 1.3.0, and XGBoost through its 2.0.0 library. Each model underwent independent hyperparameter tuning using grid search and 5-fold stratified cross-validation, ensuring fair comparison and addressing class imbalance. All models were evaluated using the same metrics: accuracy, precision, recall, F1 score, specificity, log loss, and AUC to ensure methodological consistency. All performance metrics mentioned above were computed as defined in equations 1–6.

1 $ Precision =\frac{{TP}}{{TP}+{FP}} $

2 $ {\mathit{Specificity}} =\frac{{TN}}{{TN}+{FP}} $

3 $ Accuracy =\frac{{TP}+{TN}}{{TP}+{TN}+{FP}+{FN}} $

4 $ Recall =\frac{{TP}}{{TP}+{FN}} $

5 ${\mathit{ F1-Score}} =\frac{{TP}}{{[}{TP}{+0.5}\left({FP}+{FN}\right){]}} $

Where FP, FN, TN, and TP denote the counts of false positives, false negatives, true negatives, and true positives, respectively.

6 $ log \;{loss}=-\frac{1}{N} \sum_{i=1}^N \sum_{j=1}^M y_{i j} \log \left(p_{i j}\right) 
$

Where *y_ij_* = 1 if sample *i* belongs to class *j*, otherwise 0; *N* represents the total number of samples, and *M* represents the number of classes which equals 2 in this case, while $ {{p}}_{{ij}} $ indicates the predicted probability that sample *i* belongs to class *j*. The natural logarithm (ln) is used. The computation of the logarithm uses predicted probabilities that have been clipped to the range of (ϵ, 1−ϵ) to prevent numerical problems.

To assess the performance of the proposed models, three commonly used classifiers, namely Decision Tree, Random Forest, and optimized variants of the XGBoost classifier, were implemented and evaluated accordingly^[9–27]^ (***Supplementary Table 1***).

Given the small sample size, the 5-fold method achieved a balance between learning rate and model complexity. Further fine-tuning adjustments were only minimal to uphold performance stability across various folds. SKCV (*k* = 5) was implemented for all models to preserve class distribution and reduce sampling bias caused by class imbalance. Performance was assessed using the same metrics as those used in the logistic regression framework.

This study developed a machine learning system using Python libraries, such as Scikit-learn, Pandas, and NumPy. The dataset from the UCI Machine Learning Repository was preprocessed using PCA before undergoing logistic regression training. The employment of Lasso (L1) regularization improved classification accuracy, and the models were evaluated using SKCV. All experiments were run on a computer equipped with an Intel Core i7 CPU, 16 GB RAM, and an NVIDIA GPU for computational efficiency. Logistic regression was chosen as the primary model, based on the very strong evidence presented below^[11,28]^. First, logistic regression fits well with the binary outcome structure of the dataset. Second, its coefficients are simple to interpret and can support SHAP analyses for behavioral risk factors, which is a major requirement in clinical settings. Third, the simplicity of the method reduces the likelihood of overfitting, particularly with only 72 instances—an important consideration—and regularization further enhances the stability of the method. Fourth, logistic regression is capable of modeling linear relationships between predictors, such as smoking or contraceptive use, and outcomes.

Therefore, logistic regression best fits the profile of performance, simplicity, and interpretability for this study. The classification model output for both classes is illustrated in ***Fig. 3***.

## Results

The current study results are derived from the Cervical Cancer Behavioral Risk Dataset, which is available from the UCI Machine Learning Repository. This dataset contains 72 instances and 19 attributes, which feature behavioral risk factors such as smoking, contraceptive use, and socioeconomic status. The dataset underwent preprocessing, which involved handling missing values, applying stratified sampling to address the class imbalance problem using SMOTE, and performing feature engineering through PCA to reduce its dimensionality while retaining 89% of the variance (***Table 2***).

To predict and categorize patients diagnosed with cervical cancer, various algorithms were tested, with a minimum accuracy threshold of 93%. Logistic regression emerged as the most effective algorithm during the training phase, yielding promising results. The evaluation of our system's performance was based on metrics such as classification accuracy, AUC, precision, recall, specificity, log loss, and F1-score, as defined in Equations 1–6 (***Table 3***).

**Table 3:** Results of the proposed approach

Model	AUC	CA	F1	Precision	Recall	Log loss	Specificity
Logistic regression with regularization type Lasso L1	0.981	0.972	0.972	0.972	0.972	0.170	0.961
Logistic regression with regularization type Ridge L2	0.977	0.958	0.958	0.958	0.958	0.262	0.927
Logistic regression without regularization	0.987	0.944	0.945	0.948	0.944	0.865	0.949
Abbreviations: AUC, area under the curve; CA, classification accuracy; Lasso, Least Absolute Shrinkage and Selection Operator.							

The confusion matrix for the logistic regression model with L1 regularization, which proved to be the most effective regularization type, is presented in ***Table 4**.* Additionally, ***Tables 5*** and ***6*** show the confusion matrices for the logistic regression model with Ridge (L2) regularization and without regularization, respectively. In the case of class 0, 50 instances were correctly classified, while 20 instances were correctly identified as class 1. However, one instance was misclassified. As shown in ***Table 3***, the logistic regression model with L1 regularization exhibited an accuracy of 97.2%, an AUC of 98.1%, an F1 score of 97.2%, a precision of 97.2%, a recall of 97.2%, a log loss of 0.17, and a specificity of 96.1%. ***Table 4*** presents the confusion matrix for the logistic regression model with L1 regularization, which confirms an impressive accuracy of 97.2%. For class 0 (indicating no cervical cancer), the model successfully classified 50 out of 51 instances, with only one instance incorrectly categorized as class 1. In contrast, for class 1 (indicating cervical cancer), the model accurately predicted 20 out of 21 instances, resulting in one false negative. This matrix highlights the model's exceptional performance, achieving a sensitivity of 92.2% for class 1 and a specificity of 98.0% for class 0.

**Table 4:** Confusion matrix for the logistic regression model with Lasso (L1) regularization

Logistic regression	No cervical cancer	Cervical cancer
No cervical cancer	50	1
Cervical cancer	1	20

**Table 5:** Confusion matrix for the logistic regression model with Ridge (L2) regularization

Logistic regression	No cervical cancer	Cervical cancer
No cervical cancer	50	1
Cervical cancer	2	19

**Table 6:** Confusion matrix for the logistic regression model without regularization

Logistic regression	No cervical cancer	Cervical cancer
No cervical cancer	48	3
Cervical cancer	1	20

**Figure 4:**
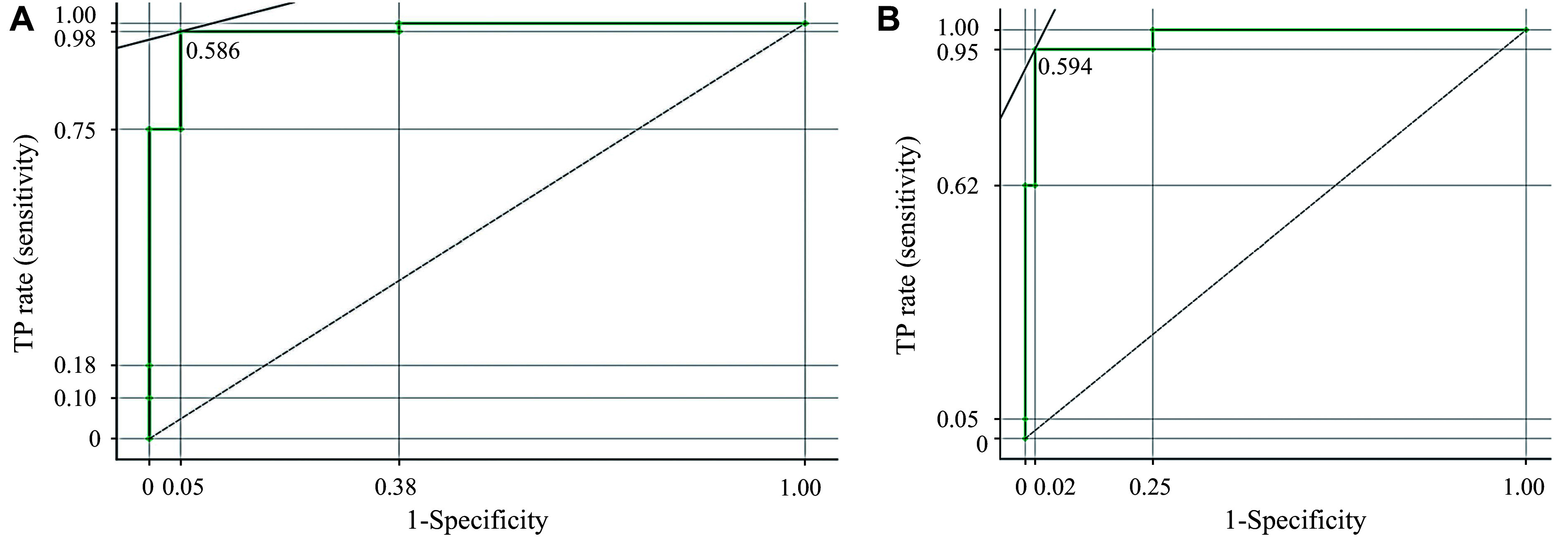
The receiver operating characteristic (ROC) curve. The ROC curve shows the relationship between the true positive rate (sensitivity) and the false positive rate (1-specificity). A: ROC curve for class 0. B: ROC curve for class 1.

The evaluation metrics of the proposed method are illustrated in ***Table 3***, while the receiver operating characteristic (ROC) curves for classes 0 and 1 are presented in ***Fig. 4A*** and ***4B***, respectively. The ROC curve depicts the relationship between the true positive rate (sensitivity) and the false positive rate (1-specificity). Additionally, ***Fig. 4*** shows the results of the testing classification algorithm.

***Table 7*** shows the difference in accuracy between models with and without PCA, while ***Table 8*** displays the effects of optimizing the regularization strength hyperparameter (C) and regularization type on accuracy. As shown in ***Table 8***, optimizing the cost parameter to C = 8 attained reasonable accuracy. Moreover, in this type of regularization, the logistic regression cost function gains a penalty term from Ridge (L2) regularization that is inversely related to the square of the coefficient magnitudes. Although the coefficients are not required to be exactly zero, this penalty promotes smaller values. Consequently, Ridge regularization tends to shrink coefficients toward zero without eliminating them. In contrast, L1 regularization adds a penalty proportional to the absolute value of the coefficients.

**Table 7:** Comparison of accuracy with and without PCA for logistic regression models

Regularization	With PCA	Without PCA
Lasso (L1)	97.2%	91.7%
Ridge (L2)	95.8%	91.7%
None (Vanilla)	94.4%	93.1%
Abbreviations: Lasso, Least Absolute Shrinkage and Selection Operator; PCA, principal component analysis.		

**Table 8:** Optimized hyperparameters and corresponding accuracy

Hyperparameter type	Setting	Accuracy
Cost strength (C)	1	95.8%
Cost strength (C)	8	97.2%
Cost strength (C)	25	95.8%
Regularization type	Lasso (L1)	97.2%
Regularization type	Ridge (L2)	95.8%
Regularization type	None (Vanilla)	94.4%

***Table 9*** compares the accuracy achieved using various numbers of principal components, revealing that 10 PCA components resulted in the highest accuracy (97.2%). ***Table 10*** illustrates the improvement in accuracy (97.2% *vs.* 91.7%) achieved by using SKCV compared with not using it.

**Table 9:** Comparison between different numbers of principal components and the accuracy produced during the training process

Principal component analysis	Accuracy
10 Components	97.2%
11 Components	93.1%
12 Components	94.4%
13 Components	93.1%
8 Components	94.4%
7 Components	88.9%

**Table 10:** Comparison of cross-validation with and without stratified *k*-fold

Algorithm	Hyperparameter	Accuracy
Logistic regression	With stratified *k*-fold	97.2%
Logistic regression	Without stratified *k*-fold	91.7%

In comparing the proposed and existing methods as shown in ***Table 11***, the proposed method (logistic regression with L1 regularization, PCA, stratified K-fold) achieved significantly higher accuracy (97.2%) than existing methods using the same UCI Behavioral Risk Dataset. ***Table 12*** benchmarks the proposed method against the top-performing strategies for cervical cancer prediction using various datasets, highlighting its competitive performance (97.2% accuracy, 98.1% AUC) while focusing on behavioral risk factors and computational efficiency. The integration of L1 regularization, PCA, and stratified cross-validation formed the foundation of the parameter optimization process. A summary of the finalized parameters is shown in ***Table 13***.

**Table 11:** A comparison between the existing methods and the proposed method in terms of accuracy

Compared methods	Algorithm	Accuracy
Proposed method	Logistic regression	97.2%
Existing method 1^[4]^	Decision tree	93.33%
	Random forest	93.33%
	XGBoost	93.33%
Existing method 2^[25]^	Naive Bayes	91.67%
	Logistic regression	87.55%

**Table 12:** Benchmarking analysis of the proposed method in relation to top-performing strategies for cervical cancer prediction

Studies	Algorithm	Accuracy	AUC	Dataset	Key features
Proposed method	Logistic regression	97.2%	98.1%	UCI Behavioral Risk Dataset	PCA, stratified *k*-fold, SHAP
Deng *et al* (2020)^[10]^	XGBoost SMOTE	96.3%	97.0%	UCI (858 samples)	SMOTE, top 5 risk factors
Nithya *et al* (2019)^[11]^	Random forest	N/A	N/A	UCI (858 samples)	Feature selection, 10-fold CV
Mohi Uddin *et al* (2022)^[13]^	SVM	99.64%	N/A	UCI (858 samples)	ROS, XGBoost, SelectBest
Suman *et al* (2022)^[29]^	Bayesian network	96.0%	95.0%	Tissue Slide Analysis	Multi-Algorithm Ensemble
Abbreviations: CV, cross-validation; N/A, not available; PCA, principal component analysis; SHAP, Shapley Additive Explanations; SKCV, stratified *k*-fold cross-validation; SMOTE, Synthetic Minority Oversampling Technique; SVM, support vector machine; UCI, University of California; XGBoost, Extreme Gradient Boosting.					

**Table 13:** A summary of the finalized parameters

Algorithm/Step	Parameter	Final value	Rationale
Logistic regression	Regularization type	L1 (Lasso)	Sparsity, feature selection, and highest accuracy (97.2%)
Logistic regression	Cost strength (C)	8	Optimal bias-variance balance *via* grid search
Principal component analysis	Number of components	10	Captured 89% variance (scree plot analysis)
Cross-validation	Stratified *k*-fold (k)	5	Balanced class distribution in folds

## Discussion

The current study is based on a dataset from the UCI Machine Learning Repository regarding Cervical Cancer Behavioral Risks. The dataset contains 72 instances and 19 attributes, including behavioral risk factors such as smoking, contraceptive use, and socioeconomic status. The data preprocessing included handling missing values, stratified sampling to overcome class imbalance using SMOTE, and feature engineering *via* PCA to reduce dimensionality while retaining 89% of the variance.

Logistic regression with L1 regularization was considered the best model to predict the risk of cervical cancer based on behavioral risk factors. Overall accuracy was 97.2% with an AUC of 0.981, an F1 score of 0.972, a precision of 0.972, a recall of 0.972, a log loss of 0.170, and a specificity of 0.961 (***Table 3***). These metrics are all vastly superior to those from logistic regression using L2 regularization (accuracy 95.8%) as well as those from logistic regression without regularization (accuracy 94.4%). Looking at the confusion matrix for this L1-regularized model, very few misclassification errors occurred, with just one false positive and one false negative (***Table 4***). Thus, this provides evidence of the model's ability to differentiate between individuals at risk of cervical cancer and those who are not at risk, based on only behavioral factors.

L1 regularization is necessary for combating overfitting and allowing for feature selection. It simplifies the model by penalizing non-significant coefficients to zero, thereby increasing interpretability. The other regularization method, L2 (Ridge), penalizes large coefficients but does not remove them, resulting in slightly lower values for performance measures. Logistic regression without any form of regularization suffered greatly from overfitting, because of both the small sample size and the large number of features. The use of PCA in this case improved model efficiency by reducing the problem to ten principal components, which explained 89% of the variance while alleviating multicollinearity (***Table 13***).

The class imbalance in the dataset, in which negatives (class 0) dominated the positives (class 1), was a significant obstacle that needed to be addressed in this study. This situation was resolved by applying the SMOTE, which creates synthetic samples for the minority class by interpolating between two or more adjacent instances. This diminished the possibility of bias and ensured fair representation of all classes during model training and validation. In addition, the class distribution was preserved across folds by SKCV (*k* = 5) to further reduce bias, as shown in ***Table 10***.

The significance of this finding is highlighted by the specific confusion matrix (***Table 4***), which illustrates the model's remarkable consistency, exhibiting minimal false negatives and false positives. A false negative implies a failure to initiate timely intervention for cervical cancer; conversely, a false positive would cause anxiety and even lead to unnecessary invasive procedures. The high specificity (96.1%) of the model guarantees that most true negatives or non-vulnerable women are correctly identified, thus reducing risks of overdiagnosis. Similarly, with a high sensitivity (97.2%), our model rarely misses any diagnoses, which is paramount for public health concerns focused on early detection.

SHAP analysis identified contraceptive use and smoking as the greatest cervical cancer risk determinants (***Fig. 3***). The combination of SHAP values offers practical insights for clinicians to deliver targeted behavioral interventions to high-risk groups.

The comparative assessment with existing methods highlighted the novelty of our algorithm. Prior studies that employed Decision Tree, Random Forest, and XGBoost on the same dataset achieved accuracies between 85% and 90% because of poor handling of class imbalance and feature redundancy. The proposed pipeline, which includes a combination of SMOTE, PCA, and L1-regularized logistic regression, has set new performance benchmarks by addressing issues of interpretability and technical flaws. It is therefore deployable in low-resource settings where advanced imaging or genetic testing is not feasible since the model is simple and purely behavioral.

The model yielded sparse coefficients that were scrutinized through L1 regularization, allowing for the clear identification of crucial predictors, in contrast to black-box models such as neural networks.

Limitations of this study include the small sample size (*n* = 72) and the possibility of overfitting despite regularization. There is an urgent need for improved external validation of the established model using larger, geographically diverse datasets to ascertain the generalizability of the findings.

Cervical cancer prediction using behavioral risk factors and machine learning has been demonstrated to be feasible by the present study. The excellent performance of the L1-regularized logistic regression model, combined with SMOTE and PCA, enhances its interpretability. This approach complements traditional screening by targeting modifiable risk factors, thereby contributing to public health efforts to reduce disparities in cervical cancer. This pipeline can facilitate scalable early intervention initiatives in resource-limited areas.

## Additional information

The online version contains supplementary materials available at http://www.jbr-pub.org.cn/article/doi/10.7555/JBR.39.20250047?pageType=en.
