# Supplementary material for: Cervical cancer perceived behavioral risk factors using logistic regression technique
Source: J Biomed Res. 2026 May 21;40(3):280–90. doi: 10.7555/JBR.39.20250047 (PMC13231354; doi:10.7555/JBR.39.20250047)
Supplement: Supplementary file 1 — The online version contains supplementary materials available at http://www.jbr-pub.org.cn/article/doi/10.7555/JBR.39.20250047?pageType=en. [file jbr-40-3-280-S1.pdf]

Available online at www.jbr-pub.org.cn

Open Access at PubMed Central

Journal of Biomedical Research, 2026 40(3): 1–1

Supplementary Data

JBR

Cervical cancer perceived behavioral risk factors using logistic regression technique

I. M. Elzein[1,2,✉], Ashraf Chamseddine[1,2], Ahmad Eltanboly[3,✉], Adam Elzein[4]

[1]Department of Electrical Engineering, College of Engineering and Technology, University of Doha for Science and Technology, Doha 24449, Qatar;
[2]Department of Environmental Health and Safety, University of Doha for Science and Technology, Doha 24449, Qatar;
[3]Faculty of Science, Galala University, New Galala City, Suez Governorate 43111, Egypt;
[4]Department of Medicine, Medical University of Lodz, Lodz 90-419, Poland.

The following configurations ensured interpretability and limited model depth to manage variance from the small dataset. Further optimized hyperparameters were implemented to achieve a balance in ensemble performance and minimize overfitting. This allowed for tuning the depth in the grid search routine.

Supplementary Table 1 **Configurations and hyperparameter settings of tree-based classifiers**

Classifier	Implementation	Key hyperparameters	Remarks
Decision Tree	sklearn.tree.DecisionTreeClassifier	Criterion: 'gini'<br>Max Depth: 4<br>Min Samples Split: 2<br>Min Samples Leaf: 1<br>Splitter: 'best'<br>Random State: 42	Ensured interpretability and controlled variance by limiting model depth due to small dataset size
Random Forest	sklearn.ensemble.RandomForestClassifier	n_estimators: 100<br>Criterion: 'gini'<br>Max Depth: 6<br>Min Samples Split: 2<br>Min Samples Leaf: 1<br>Bootstrap: True<br>Random State: 42	Hyperparameters optimized to balance ensemble performance and reduce overfitting *via* grid search
XGBoost	xgboost.XGBClassifier	Booster: 'gbtree'<br>Learning Rate (eta): 0.1<br>Max Depth: 5<br>Subsample: 0.8<br>Colsample_bytree: 0.8<br>Gamma: 0<br>n_estimators: 100<br>Regularization (L2): lambda = 1<br>Random State: 42	Tuned for efficient gradient boosting with subsampling and L2 regularization to improve generalization

✉Corresponding authors: I. M. Elzein, E-mail: 60101973@udst.edu.qa, ORCID: 0000-0002-7949-209X; Ahmad Eltanboly, E-mail: Ahmed.Eltanbolyy@gu.edu.eg, ORCID: 0000-0002-7675-3848.

Received: 05 February 2025; Revised: 07 August 2025; Accepted: 09 August 2025; Available online: 20 August 2025; Published date: 21 May 2026

CLC number: R737.33, Document code: A

The authors reported no conflict of interests.

This is an open access article under the Creative Commons Attribution (CC BY 4.0) license, which permits others to distribute, remix, adapt and build upon this work, for commercial use, provided the original work is properly cited.

© 2026 by Journal of Biomedical Research.

https://doi.org/10.7555/JBR.39.20250047
